# MiR-143 and MiR-145 Regulate IGF1R to Suppress Cell Proliferation in Colorectal Cancer

**DOI:** 10.1371/journal.pone.0114420

**Published:** 2014-12-04

**Authors:** Jiaojiao Su, Hongwei Liang, Weiyan Yao, Nan Wang, Suyang Zhang, Xin Yan, Hui Feng, Wenjing Pang, Yanbo Wang, Xueliang Wang, Zhen Fu, Yanqing Liu, Chihao Zhao, Junfeng Zhang, Chen-Yu Zhang, Ke Zen, Xi Chen, Yalei Wang

**Affiliations:** 1 Department of Gastroenterology, The First Affiliated Hospital of Anhui Medical University, Hefei, Anhui 230022, China; 2 Jiangsu Engineering Research Center for microRNA Biology and Biotechnology, State Key Laboratory of Pharmaceutical Biotechnology, School of Life Sciences, Nanjing University, 22 Hankou Road, Nanjing, Jiangsu 210093, China; 3 Department of Gastroenterology, Ruijin Hospital, Shanghai Jiaotong University School of Medicine, Shanghai 200025, China; 4 The Comprehensive Cancer Center of Drum Tower Hospital affiliated to Medical School of Nanjing University & Clinical Cancer Institute of Nanjing University, Nanjing, Jiangsu 210008, China; University of Houston, United States of America

## Abstract

Insulin-like growth factor 1 receptor (IGF1R) is a transmembrane receptor that is activated by insulin-like growth factor 1 (IGF-1) and by a related hormone called IGF-2. It belongs to the large class of tyrosine kinase receptors and plays an important role in colorectal cancer etiology and progression. In this study, we used bioinformatic analyses to search for miRNAs that potentially target IGF1R. We identified specific target sites for miR-143 and miR-145 (miR-143/145) in the 3′-untranslated region (3′-UTR) of the IGF1R gene. These miRNAs are members of a cluster of miRNAs that have been reported to exhibit tumor suppressor activity. Consistent with the bioinformatic analyses, we identified an inverse correlation between miR-143/145 levels and IGF1R protein levels in colorectal cancer tissues. By overexpressing miR-143/145 in Caco2, HT29 and SW480 colorectal cancer cells, we experimentally validated that miR-143/145 directly recognizes the 3′-UTR of the IGF1R transcript and regulates IGF1R expression. Furthermore, the biological consequences of the targeting of IGF1R by miR-143/145 were examined by cell proliferation assays *in* *vitro*. We demonstrated that the repression of IGF1R by miR-143/145 suppressed the proliferation of Caco2 cells. Taken together, our findings provide evidence for a role of the miR-143/145 cluster as a tumor suppressor in colorectal cancer through the inhibition of IGF1R translation.

## Introduction

Colorectal cancer is the third most commonly diagnosed cancer in the world, and it is more prevalent in developed countries [1]. It is estimated that the crude mean incidence of Colorectal cancer in South East Asia for both sexes was 6.95/100000 population in 2008 and the incidence increased with age [2]. At the molecular level, colorectal cancer arises from a series of genetic and epigenetic alterations that inactivate tumor suppressor genes and activate oncogenes [3]. However, the basic mechanisms underlying colorectal cancer initiation and progression remain largely unknown.

Insulin-like growth factor 1 receptor (IGF1R), a tyrosine kinase receptor for IGF-1 and IGF-2, is frequently overexpressed in tumors and has been well documented in cell culture, animal studies and humans to play a role in malignant transformation, progression, protection from apoptosis, and metastasis [4], [5]. The IGF receptor family consists of three transmembrane proteins, and the IGF1R gene is located on chromosome 15q26, which encodes a single polypeptide of 1367 amino acids that is constitutively expressed in most cells [5]. IGF1R activation leads to autophosphorylation on tyrosines 1131, 1135 and 1136 in the kinase domain, followed by phosphorylation of juxtamembrane tyrosines and carboxy-terminal serines [6]. This is followed by recruitment of specific docking intermediates, including insulin-receptor substrate-1 (IRS-1), Shc and 14-3-3 proteins [7], [8]. These molecules link the IGF1R to diverse signaling pathways, allowing the induction of growth, transformation, differentiation and protection against apoptosis [9]. Upon IGF-1 binding, IGF1R activates the PI3K/Akt cascade, which promotes G1 to S cell cycle progression and elevates cell proliferation [10], [11]. IGF1R is overexpressed during colorectal carcinogenesis, with the highest expression observed in the proliferating cells at the base of the colonic crypts [11], [12]. However, the role of IGF1R in colorectal cancer remains to be elucidated.

Over the past decade, a novel class of small RNA molecules known as microRNAs (miRNAs) has emerged as major regulators of the initiation and progression of human cancers, including colorectal cancer [13]–[15]. miRNAs are small (∼22-nucleotide long), single-stranded noncoding RNA molecules that negatively regulate gene expression by binding to the 3′-untranslated region (3′-UTR) of target mRNA molecules, which results in either degradation of the transcript or inhibition of translation [16]–[18]. Many miRNAs work in conjunction with one another to fine tune protein expression on a global level. Thus, miRNAs play a significant role in post-transcriptional gene regulation. Importantly, recent studies indicate that the dysregulation of miRNAs is associated with human malignancies and suggest a causal role of miRNAs in cancer etiology [19]. miRNAs presumably play a role in cancer because they can affect the translation and stability of targeted oncogenes or tumor suppressors, which ultimately influence cellular physiology [19], [20]. For example, miR-143 and miR-145 (miR-143/145), which are located in a cluster within the 5q32–33 chromosomal region, are downregulated in many types of cancers, including colorectal cancer [21], [22]. miR-143 and miR-145 have been shown to possess anti-tumorigenic activity, including involvement in various cancer-related processes such as proliferation, invasion and migration [23].

Although dysregulation of IGF1R and miRNAs is associated with tumorigenesis in human colorectal cancer, little is known about miRNAs that act on IGF1R. In this study, we found that IGF1R was directly regulated by miR-143 and miR-145 in colorectal cancer cells. Furthermore, we showed that miR-143/145 can inhibit IGF1R expression to suppress the proliferation of colorectal cancer cells.

## Materials and Methods

### Human tissue

Colorectal cancer tissues and paired adjacent noncancerous tissues were obtained from patients undergoing surgical procedures at The First Affiliated Hospital of Anhui Medical University (Hefei, China). Both tumors and noncancerous tissues were subjected to histological analysis for diagnostic confirmation. The pathological type of each cancer was identified as adenocarcinoma. Written consent was provided by all of the patients or their guardians, and the Ethics Committee of The First Affiliated Hospital of Anhui Medical University approved all aspects of this study. Tissue fragments were immediately frozen in liquid nitrogen at the time of surgery and stored at –80°C. The clinical features of the patients are listed in Table S1in File S1.

### Cell culture

The human colorectal cancer cell lines Caco2, HT29 and SW480 were purchased from the Shanghai Institute of Cell Biology, Chinese Academy of Sciences (Shanghai, China). Caco2 cells were cultured in DMEM (Gibco, Carlsbad, CA, USA) supplemented with 10% fetal bovine serum (Gibco) in a humidified incubator at 37°C with 5% CO_2_; HT29 and SW480 cells were cultured in RPMI-1640 (Gibco, Carlsbad, CA, USA) supplemented with 10% fetal bovine serum (Gibco) in a humidified incubator at 37°C with 5% CO_2_.

### RNA isolation and quantitative RT-PCR

Total RNA was extracted from the cultured cells and tissues using Trizol Reagent (Invitrogen) according to the manufacturer’s instructions. Assays to quantify mature miRNAs were performed using TaqMan microRNA probes (Applied Biosystems, Foster City, CA) according to the manufacturer’s instructions. Briefly, 1 µg of total RNA was reverse-transcribed to cDNA using AMV reverse transcriptase (TaKaRa, Dalian, China) and a stem-loop RT primer, and one tenth of cDNA was used per qPCR reaction (Applied Biosystems). The reaction conditions were as follows: 16°C for 30 min, 42°C for 30 min and 85°C for 5 min. Real-time PCR was performed using a TaqMan PCR kit and an Applied Biosystems 7300 Sequence Detection System (Applied Biosystems). The reactions were incubated in a 96-well optical plate at 95°C for 5 min, followed by 40 cycles of 95°C for 15 s and 60°C for 1 min. All of the reactions were run in triplicate. After the reactions were complete, the cycle threshold (C_T_) data were determined using fixed threshold settings, and the mean C_T_ was determined from triplicate PCRs. A comparative C_T_ method was used to compare each condition to the control reactions. U6 snRNA was used as an internal control, and the relative amount of miRNA normalized to U6 was calculated with the equation 2^−ΔΔCT^, in which ΔΔC_T_ = (C_T miRNA_–C_T U6_)_target_–(C_T miRNA_–C_T U6_)_control_.

To quantify IGF1R and GAPDH mRNA, 1 µg of total RNA was reverse-transcribed to cDNA using Oligo d(T)18 primers (TaKaRa) and ThermoScript reverse transcriptase (Invitrogen). The reaction conditions were as follows: 42°C for 60 min and 70°C for 10 min. Real-time PCR was then performed with the RT product, SYBR Green dye (Invitrogen) and specific primers for IGF1R and GAPDH. The sequences of the primers were as follows: IGF1R (sense): 5′-GCCAAGCTAAACCGGCTAAA-3′; IGF1R (antisense): 5′-TATCCTGTTTTGGCCTGGACATA-3′; GAPDH (sense): 5′-GATATTGTTGCCATCAATGAC-3′; and GAPDH (antisense): 5′-CGCTCATTGCCGATAGTG-3′. The reactions were incubated at 95°C for 5 min, followed by 40 cycles of 95°C for 30 s, 55°C for 30 s and 72°C for 1 min. After the reactions were complete, the C_T_ values were determined by setting a fixed threshold. The relative amount of IGF1R mRNA was normalized to GAPDH.

### MiRNA overexpression

miRNA overexpression was achieved by transfecting cells with a miRNA mimic, a synthetic RNA oligonucleotide duplex mimicking the miRNA precursor. Synthetic RNA molecules, including pre-miR-143, pre-miR-145 and a scrambled negative control RNA (pre-miR-control), were purchased from GenePharma (Shanghai, China). Caco2, HT29 and SW480 cells were seeded in 6-well plates and transfected using Lipofectamine 2000 (Invitrogen) on the following day when the cells were approximately 70% confluent. For the miRNA overexpression experiments, 100 pmol of pre-miR-143, 100 pmol of pre-miR-145 or 50 pmol of both pre-miR-143 and pre-miR-145 were used. At 6 h after transfection, the medium for the Caco2 cells was changed to DMEM supplemented with 2% fetal bovine serum, and the medium for the HT29 and SW480 cells was changed to RPMI-1640 supplemented with 2% fetal bovine serum. The cells were harvested at 24 h or 48 h post-transfection for the isolation of total RNA or protein, respectively.

### Plasmid construction and siRNA interference assay

A mammalian expression plasmid (pReceiver-M02-IGF1R) designed to specially express the full-length open reading frame (ORF) of human IGF1R without the miR-143/145-responsive 3′-UTR was purchased from GeneCopoeia (Germantown, MD, USA). An empty plasmid served as a negative control. The siRNA (sense: 5′-GCGGCUGGAAACUCUUCUATT-3′; antisense: 5′-UAGAAGAGUUUCCAGCCGCTT-3′) targeting human IGF1R was designed and synthesized by Invitrogen (Carlsbad, CA, USA). A scrambled siRNA (Invitrogen) was included as a negative control. The overexpression plasmid or siRNA were transfected into Caco2 cells using Lipofectamine 2000 (Invitrogen) according to the manufacturer’s instructions. Total RNA or protein was isolated 24 h or 48 h after transfection. IGF1R mRNA and protein expression levels were assessed by quantitative RT-PCR and Western blotting.

### Luciferase reporter assay

The entire 3′-UTR of human IGF1R was amplified by PCR using human genomic DNA as a template. The PCR products were then inserted into the p-MIR-reporter plasmid (Ambion, Austin, TX, USA). The insertion was confirmed to be correct by DNA sequencing. To test the binding specificity, the sequences that interact with the seed sequence of miR-143 and miR-145 were mutated (from UUGGGAG to AACCCUC for the miR-143 binding site and from UUGGGAG to AACCCUC for the miR-145 binding site), and the mutant IGF1R 3′-UTR was inserted into an equivalent luciferase reporter plasmid. For luciferase reporter assays, Caco2, HT29 and SW480 cells were seeded in 24-well plates and co-transfected with 0.8 µg of firefly luciferase reporter plasmid, 0.8 µg of β-galactosidase (β-gal) expression plasmid (Ambion), and equal amounts (20 pmol) of miR-143/145 mimic or a scrambled negative control RNA using Lipofectamine 2000 (Invitrogen). The β-gal plasmid was used as a transfection efficiency control. Cells were harvested 24 h after transfection and analyzed for luciferase activity using a luciferase assay kit (Promega, Madison, WI, USA).

### Protein isolation and Western blotting

Cells or tissues were lysed in a RIPA lysis buffer (50 mM Tris-HCl, 150 mM NaCl, 0.1% SDS, 1% NP-40, 0.25% sodium deoxycholate and 1 mM EDTA, pH 8.0) with freshly added protease inhibitor cocktail (Roche) for 30 min on ice and were then centrifuged at 16,000×g at 4°C for 10 min. The supernatant was collected, and the protein concentration was calculated with a BCA protein assay kit (Thermo Scientific, Rockford, IL, USA). Proteins were separated by SDS-PAGE (Bio-Rad). After electrophoresis, the proteins were electrotransferred to PVDF membranes (Bio-Rad) and then blocked with 5% skim milk for 1 h. The membranes were then incubated with primary antibodies at 4°C for 12 h. After three washes in TBST, the membranes were incubated with horseradish peroxidase-conjugated secondary antibody for 1 h at room temperature. After three washes, the membranes were incubated with the SuperSignal West Pico chemiluminescence substrate (Pierce). The same membrane was also probed with a GAPDH antibody to serve as a loading control. IGF1R and GAPDH antibodies were purchased from Cell Signaling (#3018) and Santa Cruz Biotechnology (sc-25778), respectively.

### Cell proliferation assay

Caco2 cells were plated at 5×10^4^ cells per well in 96-well plates and then incubated overnight in DMEM supplemented with 10% FBS. Cells were collected at 12, 24, 48 and 72 h post-transfection. After transfection, 10 µL of Cell Counting Kit-8 (#C0038, Beyotime) was added into the corresponding test well and incubated for 1 h. Absorbance was measured at a wavelength of 450 nm.

### EdU proliferation assay

To assess cell proliferation, Caco2 cells were seeded in 24-well plates. The cells were incubated under standard conditions in complete media. Transfection of the cells was performed the following day as described above. Forty-eight hours after transfection, cell proliferation was detected using the incorporation of 5-ethynyl-2′-deoxyuridine (EdU) with the EdU Cell Proliferation Assay Kit (Ribobio, Guangzhou, China). Briefly, the cells were incubated with 50 µM EdU for 3 h before fixation, permeabilization and EdU staining, which were performed according to the manufacturer’s protocol. The cell nuclei were stained with DAPI (Sigma) at a concentration of 1 µg/mL for 10 min. The proportion of cells that incorporated EdU was determined by fluorescence microscopy.

### Statistical analysis

All of the Western blotting and proliferation assay images are representative of at least three independent experiments. Quantitative RT-PCR and luciferase reporter assays were performed in triplicate, and each individual experiment was repeated several times. The results are presented as the means ± SE of at least three independent experiments. Observed differences were considered statistically significant at p<0.05 using Student’s t-test.

## Results

### Identification of conserved miR-143 and miR-145 target sites within the 3′-UTR of IGF1R

**Figure 1:**
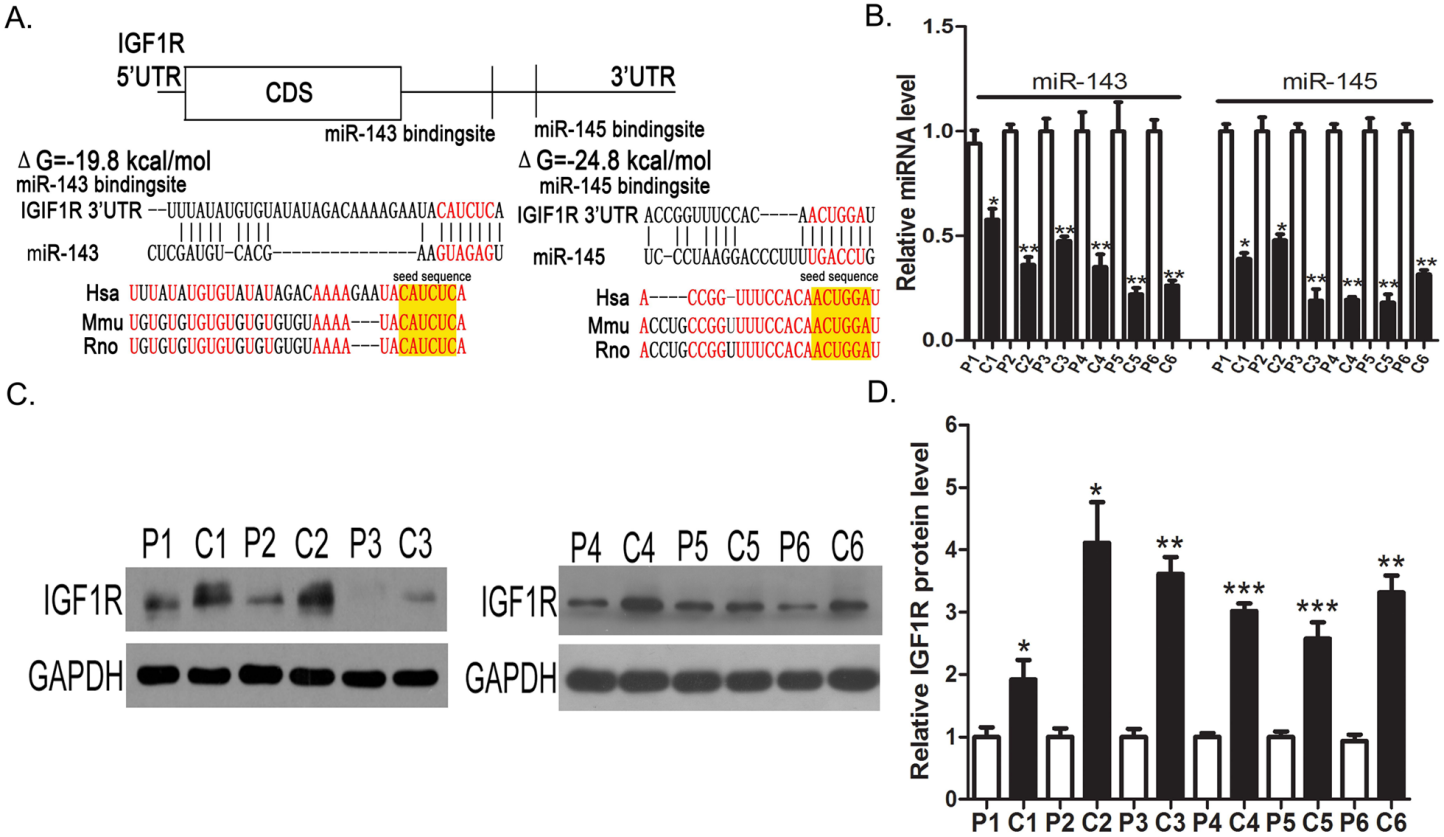
Identification of an inverse correlation between miR-143/145 levels and IGF1R protein levels in human colorectal cancer tissues. (**A**) Schematic description of the hypothetical duplexes formed by the interactions between the binding sites in the IGF1R 3′-UTR (top) and miR-143/145 (bottom). The predicted free energy value of each hybrid is indicated. The seed recognition sites are denoted, and all nucleotides in these regions are highly conserved across several species, including human, mouse and rat. (**B**) Quantitative RT-PCR analysis of miR-143 and miR-145 levels in six pairs of colorectal cancer tissue (C) and noncancerous tissue (P) samples. (**C and D**) Western blot analysis of IGF1R protein levels in six pairs of C and P samples. C: representative image; D: quantitative analysis. *P<0.05; **P<0.01.

Three computational algorithms, including TargetScan [24] and RNAhydrid [25], were used in combination to identify potential miRNAs that target IGF1R. All three algorithms predicted miR-143 and miR-145 as regulators of IGF1R. The predicted interactions between miR-143/145 and the target sites within the 3′-UTR of IGF1R are illustrated in Figure 1A. One predicted hybridization was observed between both miR-143 and miR-145 and the 3′-UTR of IGF1R. Although the two target sites within the 3′-UTR of IGF1R were near one another, the sites were non-overlapping. There was perfect complementarity between the seed region (the core sequence that encompasses the first 2–8 bases of the mature miRNA) and the cognate targets. The minimum free energy values of the hybridizations between miR-143 and IGF1R and between miR-145 and IGF1R were –19.8 and –24.8 kcal/mol, respectively, which are well within the range of genuine miRNA-target pairs. Furthermore, the miR-143/145 binding sequences in the IGF1R 3′-UTR were highly conserved across species. Thus, miR-143 and miR-145 were selected as candidates for further experimental verification.

### Identification of an inverse correlation between miR-143/145 levels and IGF1R protein levels in colorectal cancer tissues

Interest in the miR-143/145 cluster and IGF1R was also supported by the recent identification of these two miRNAs as candidate tumor suppressors [26] and IGF1R as an oncogene in colorectal cancer [8]. We next investigated whether miR-143/145 levels were inversely correlated with IGF1R protein expression in colorectal cancer tissues. After determining the levels of miR-143/145 and the protein levels of IGF1R in six pairs of colorectal cancer tissues and corresponding noncancerous tissues, we showed that miR-143 and miR-145 levels were consistently downregulated in colorectal cancer tissues (Figure 1B), whereas IGF1R protein levels were dramatically higher in the colorectal cancer tissues (Figure 1C and 1D). These results indicated that miR-143/145 expression levels are inversely correlated to IGF1R protein expression in human colorectal cancer tissues.

### Validation of IGF1R as a direct target of miR-143/145

**Figure 2:**
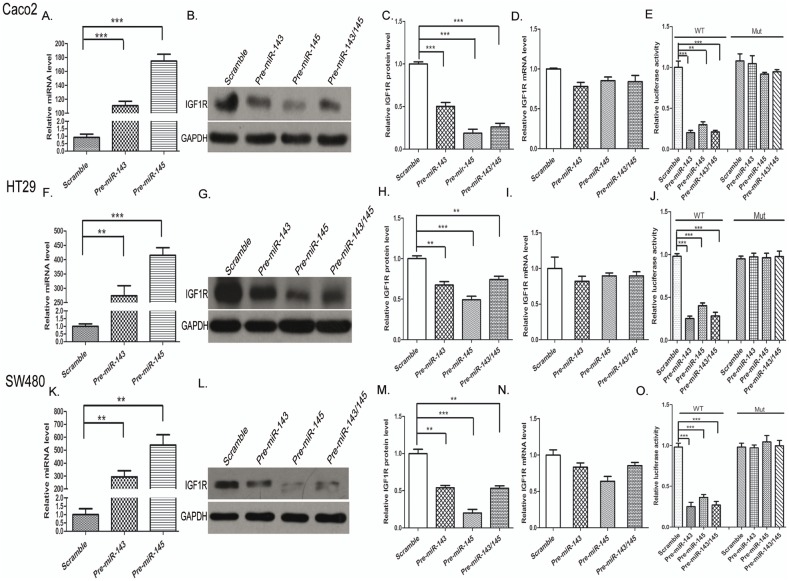
miR-143/145 directly regulate IGF1R expression at the post-transcriptional level. (**A, F and K**) Quantitative RT-PCR analysis of miR-143/145 levels in Caco2, HT29 and SW480 cells treated with a scrambled control, pre-miR-143, pre-miR-145 or both pre-miR-143 and pre-miR-145. (**B, C; G, H; and L, M**) Western blot analysis of IGF1R protein levels in Caco2 cells treated with a scrambled control, pre-miR-143, pre-miR-145 or both pre-miR-143 and pre-miR-145. B, G and L: representative image; C, H and M: quantitative analysis. (**D, I and N**) Quantitative RT-PCR analysis of IGF1R mRNA levels in Caco2, HT29 and SW480 cells treated with a scrambled control, pre-miR-143, pre-miR-145 or both pre-miR-143 and pre-miR-145. (**E, J and O**) Direct recognition of the IGF1R 3′-UTR by miR-143/145. Caco2, HT29 and SW480 cells were co-transfected with firefly luciferase reporters containing either wild-type (WT) or mutant (MUT) miR-143/145 binding sites in the IGF1R 3′-UTR and a scrambled control, pre-miR-143, pre-miR-145 or both pre-miR-143 and pre-miR-145. Twenty-four hours after transfection, the cells were assayed using a luciferase assay kit. The results are displayed as the ratio of firefly luciferase activity in the miR-143/145-transfected cells to the activity in the control cells. *P<0.05; **P<0.01.

The correlation between miR-143/145 and IGF1R was further examined by evaluating IGF1R expression in the human colorectal cancer cell lines Caco2, HT29 and SW480 after overexpression of miR-143/145. In these experiments, overexpression was achieved by transfecting Caco2, HT29 and SW480 cells with pre-miR-143 and/or pre-miR-145, which are synthetic RNA oligonucleotides that mimic the miR-143 and miR-145 precursors. The efficient overexpression of miR-143/145 is shown in Figure 2A, 2F and 2K. As anticipated, overexpression of miR-143 and/or miR-145 significantly suppressed IGF1R protein levels in Caco2, HT29 and SW480 cells (Figure 2B, 2C; 2G, 2H; and 2L, 2M). To determine the regulatory level at which miR-143/145 influenced IGF1R expression, we repeated the above experiments and examined the expression of IGF1R mRNA after transfection. Overexpression of miR-143/145 did not affect IGF1R mRNA stability (Figure 2D, 2I and 2N). These results demonstrated that miR-143/145 specifically regulates IGF1R expression at the post-transcriptional level, which is the most common mechanism for animal miRNAs.

To determine whether the negative regulatory effects of miR-143/145 on IGF1R expression were mediated through the binding of miR-143/145 to the predicted target sites in the 3′-UTR of the IGF1R mRNA, the full length 3′-UTR of IGF1R containing the two predicted miR-143/145 binding sites was inserted downstream of the firefly luciferase gene in a reporter plasmid. The resulting plasmid was transfected into Caco2, HT29 and SW480 cells along with a transfection control plasmid (β-gal) and either pre-miR-143/145 or scrambled negative control RNAs. As expected, the luciferase activity was markedly reduced in cells transfected with pre-miR-143 and/or pre-miR-145 (Figure 2E, 2J and 2O). Furthermore, we introduced point mutations into the corresponding complementary sites in the 3′-UTR of IGF1R to eliminate the predicted miR-143/145 binding sites. This mutated luciferase reporter was unaffected by overexpression of miR-143/145 (Figure 2E, 2J and 2O). This finding suggested that the miRNA binding sites strongly contributed to the miRNA:mRNA interaction that mediated the post-transcriptional repression of IGF1R expression. In conclusion, our results demonstrated that miR-143/145 directly recognizes and binds to the 3′-UTR of the IGF1R mRNA transcript to suppress IGF1R expression in colorectal cancer cells.

### MiR-143 and miR-145 can suppresses proliferation in colorectal cancer cells

**Figure 3:**
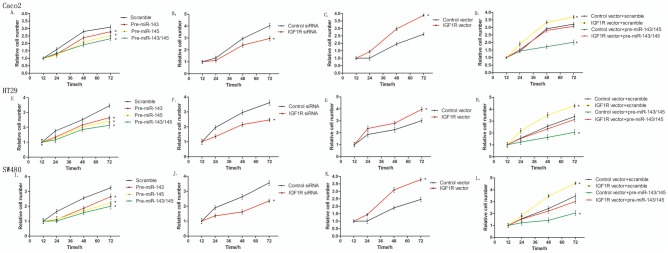
The effect of miR-143/145 on the proliferation of colorectal cancer cells. (**A, E and I**) CCK-8 viability assays were performed 12, 24, 48 and 72 h after the transfection of Caco2 (A), HT29 (E) and SW480 (I) cells with a scrambled control, pre-miR-143, pre-miR-145 or both pre-miR-143 and pre-miR-145. (**B, F and J**) CCK-8 viability assays were performed 12, 24, 48 and 72 h after the transfection of Caco2 (B), HT29 (F) and SW480 (J) cells with either a scrambled control siRNA or an IGF1R siRNA. (**C, G and K**) CCK-8 viability assays were performed 12, 24, 48 and 72 h after the transfection of Caco2 (C), HT29 (G) and SW480 (K) cells with either a control vector or an IGF1R overexpression vector. (**D, H and L**) CCK-8 viability assays were performed 12, 24, 48 and 72 h after the transfection of Caco2 (D), HT29 (H) and SW480 (L) cells with either a scrambled control, pre-miR-143/145 or both pre-miR-143/145 and the IGF1R overexpression vector. *P<0.05; **P<0.01.

We next focused on studying the roles of miR-143/145 in IGF1R regulation. Because IGF1R is essential for the regulation of proliferation and cell cycle progression, we evaluated the effects of miR-143/145 on colorectal cancer cell proliferation using a CCK-8 assay. In support of the notion that miR-143/145 function as key cancer suppressed miRNAs [26], Caco2, HT29 and SW480 cells transfected with pre-miR-143/145 showed suppressed proliferation (Figure 3A, 3E and 3I). Furthermore, we assessed the role of IGF1R on cell proliferation. To knock down IGF1R, an siRNA targeting IGF1R was designed and transfected into Caco2, HT29 and SW480 cells. To overexpress IGF1R, a plasmid expressing the IGF1R ORF was transfected into Caco2, HT29 and SW480 cells. The efficient overexpression or knockdown of IGF1R is shown in Figure S1 in File S1. Consistent with previous studies showing that IGF1R promotes cell proliferation [27], Caco2, HT29 and SW480 cells transfected with the IGF1R overexpression plasmid proliferated at a significantly higher rate (Figure 3C, 3G and 3K), whereas IGF1R knockdown with the siRNA significantly suppressed proliferation (Figure 3B, 3F and 3J). Thus, the anti-proliferative effect of IGF1R knockdown was similar to miR-143/145 overexpression. Furthermore, we constructed a IGF1R overexpression plasmid resistant to miR-143/145 (by elimination of its 3′-UTR). Compared to cells transfected with pre-miR-143/145, the cells transfected with pre-miR-143/145 and the IGF1R overexpression plasmid exhibited significantly higher proliferation rates (Figure 3D, 3H and 3L), suggesting that miR-143/145–resistant IGF1R rescued the suppression of IGF1R by miR-143/145 and attenuated the anti-proliferative effect of miR-143/145.

**Figure 4:**
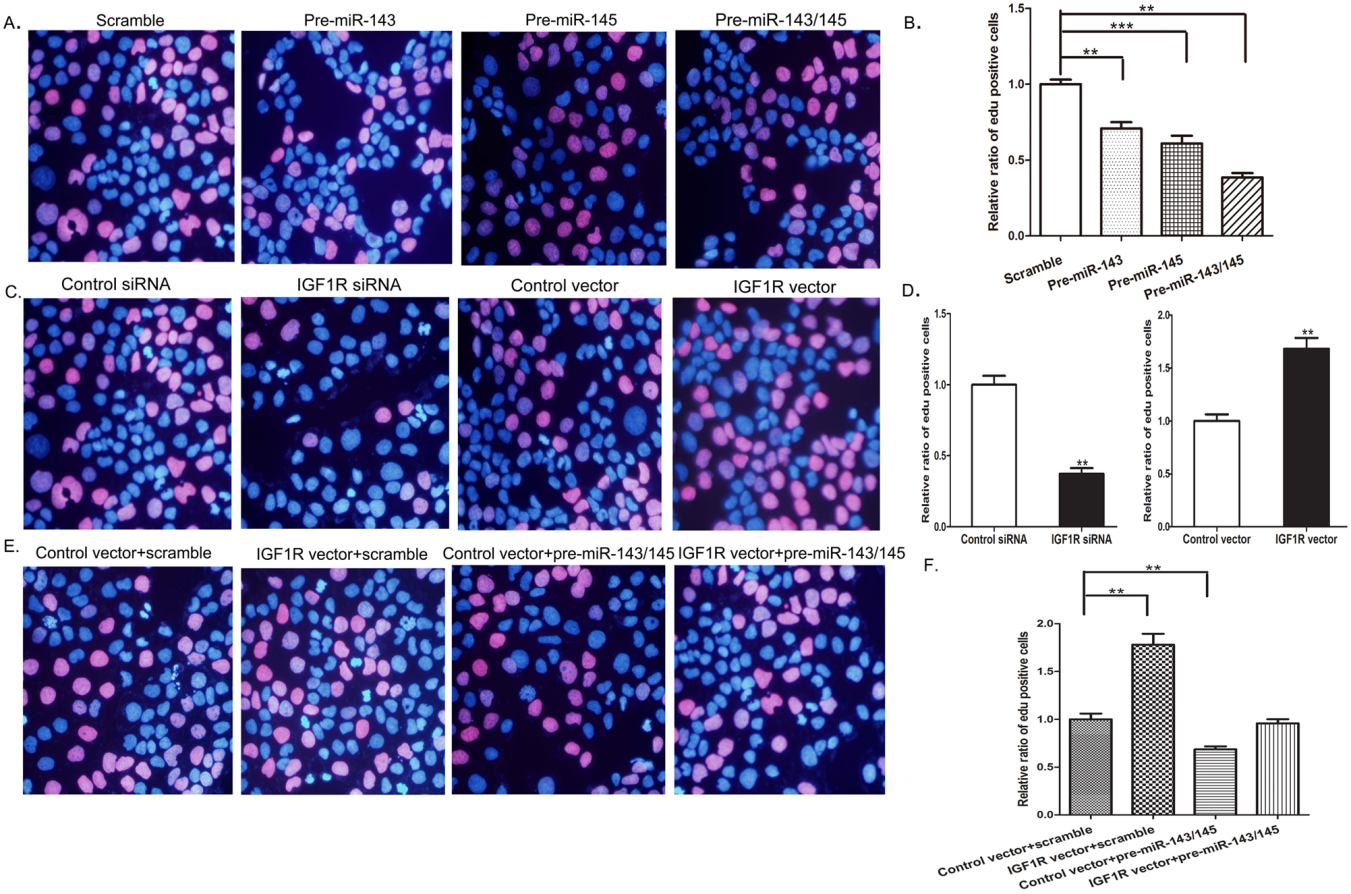
EdU proliferation assay analysis of the effect of IGF1R-targeted miR-143/145 on the growth of colorectal cancer cells. The red fluorescent cells are in the S phase of mitosis, and the blue fluorescent cells represent all of the cells. (**A and B**) The EdU proliferation assay was performed 48 h after the transfection of Caco2 cells with a scrambled control, pre-miR-143, pre-miR-145 or both pre-miR-143 and pre-miR-145. A: representative image; B: ratio of EdU-positive Caco2 cells. (**C and D**) The EdU proliferation assay was performed 48 h after the transfection of Caco2 cells with a scrambled control siRNA, IGF1R siRNA, control vector or the IGF1R overexpression vector. C: representative image; D: ratio of EdU-positive Caco2 cells. (**E and F**) The EdU proliferation assay was performed 48 h after the transfection of Caco2 cells with a scrambled control plus control vector, a scrambled control plus IFG1R overexpression vector, pre-miR-143/145 plus control vector, or pre-miR-143/145 plus IFG1R overexpression vector. E: representative image; F: ratio of EdU-positive Caco2 cells. *P<0.05; **P<0.01.

To further test the biological effect of IGF1R-targeted miR-143/145 on the growth of colorectal cancer cells, the effect of miR-143/145 and IGF1R on cell proliferation was examined with an EdU assay, an immunochemical detection method that measures nucleotide analogue incorporation into newly replicated DNA. Consistent with the results from the CCK-8 assay, the percentage of EdU-positive cells was significantly lower in cells transfected with pre-miR-143/145 (Figure 4A and 4B). Similarly, significantly more EdU-positive cells were observed in the cells with IGF1R overexpression, whereas IGF1R-siRNA transfected cells showed the opposite effect on cell proliferation (Figure 4C and 4D). These results again demonstrated that decreased IGF1R levels yielded the same phenotype observed by miR-143/145 overexpression. To further examine the functional relationship between miR-143/145 and IGF1R, Caco2 cells were simultaneously transfected with pre- miR-143/145 and the IGF1R overexpression plasmid. As expected, overexpression of IGF1R dramatically rescued the suppressive effect of miR-143/145 on cell proliferation (Figure 4E and 4F). Taken together, the results demonstrate that miR-143/145 suppress cell proliferation by silencing IGF1R.

## Discussion

Over the past decades, major advances in our comprehension of colorectal cancer biology have led to improved diagnostic and prognostic techniques and to the development of novel targeted therapies. However, the efficacy of new treatments remains limited by a combination of drug resistance and by our limited understanding of tumor cell signaling pathways. Recently, an important role for IGF1R in the genesis and progression of colorectal cancer has emerged [28]–[30]. Thus, IGF1R offers a particularly promising molecular target for colorectal cancer therapy. However, very little is known about the regulation of IGF1R expression in colorectal cancer. Thus, the molecular mechanisms underlying IGF1R regulatory pathways need to be fully elucidated.

In this study, we predicted IGF1R to be a target of miR-143 and miR-145, which are a cluster of miRNAs that have been reported in many studies to be downregulated and to function as tumor suppressors in most cancers [21], [22]. After measuring the expression levels of miR-143/145 and IGF1R in human colorectal cancer tissue and paired noncancerous tissue, we detected an inverse correlation between miR-143/145 levels and IGF1R protein levels. Furthermore, by overexpressing miR-143/145 in Caco2 cells, we experimentally validated that miR-143/145 directly inhibited IGF1R translation. Finally, we showed that miR-143/145 inhibited IGF1R expression and consequently suppressed the proliferation of colorectal cancer cells *in* *vitro*. The results delineate a novel regulatory network employing miR-143/145 and IGF1R to fine-tune cell proliferation. We also provided evidence that restoration of IGF1R expression can reverse miR-143/145-suppressed cell proliferation. Interestingly, it was previously shown that miR-145 can target insulin receptor substrate-1 (IRS-1) and IGF1R in colon cancer cells [31]–[33]. However, while an IRS-1 resistant to miR-145 can rescue colon cancer cells from miR-145-induced growth inhibition, an IGF1R resistant to miR-145 failed to rescue colon cancer cells from growth inhibition [33]. This is not in accord with our findings. It seems that whether miR-145 can target IGF1R to regulate cell growth is dependent on the cell and tumor types. Under different circumstances, miR-145 may exert different functions. Nevertheless, the findings of ours and others highlight that miR-143/145 may act as tumor-suppressive miRNAs, and that the targeting of IGF1R may be one mechanism by which the miR-143/145 cluster exerts its tumor suppressive function. Therefore, the modulation of IGF1R by miR-143/145 may explain why the downregulation of miR-143/145 during colorectal carcinogenesis can promote cancer progression.

miRNAs that are located nearby in the genome (within 10 kb) are considered to belong to a single cluster. miRNA clusters are transcribed coordinately as polycistronic units that are processed to produce the individual miRNA members, which result in co-expression of the miRNAs. miRNAs originating from a single cluster often display corresponding sequence homology (e.g., miR-15a and miR-16) and, as a result, possess overlapping targets. However, miR-143 and miR-145 do not share sequence homology making their individual and combined functions less clear. The target genes of miR-143 identified and verified thus far are primarily MAPK signaling molecules, such as ERK5 and KRAS [34], [35], whereas target genes of miR-145 are drastically different, including Mucin-1 and RTKN [36], [37]. One goal of this study was to determine whether miR-143 and miR-145 function individually or cooperatively. In our study, bioinformatic analyses of the 3′-UTR of IGF1R revealed two non-overlapping target elements for miR-143 and miR-145. Experimental validations supported the hypothesis that miR-143 and miR-145 exhibited a cooperative repression of IGF1R expression. Thus, our results provide novel evidence that individual miRNAs within a cluster that display a co-expression pattern but lack sequence homology can simultaneously and cooperatively repress a given target mRNA.

miRNAs are frequently aberrantly expressed or mutated in cancer, which suggests a role for miRNAs as a novel class of oncogenes or tumor suppressor genes [19], [20]. Given the involvement of miRNAs in cancer development, the manipulation of cellular miRNA levels has emerged as a potential therapeutic strategy. Efforts to predictably alter intracellular transcript profiles by increasing specific miRNA levels through either transfection or viral delivery methods have demonstrated the potential of this strategy to modulate cellular physiology [38]. Conversely, attempts to reduce miRNA levels using biologically stable antisense oligonucleotides, such as antagomirs, have also proven capable of altering intracellular transcript profiles [39]. In this study, miR-143 and miR-145 showed an anti-tumor effect *in* *vitro* through the negative regulation of IGF1R in human colorectal cancer. We hypothesize that a combination replacement treatment with both miR-143 and miR-145 will be a promising strategy for cancers exhibiting downregulation of miR-143 and miR-145. In future studies, an effective drug delivery system needs to be developed for the use of miR-143 and miR-145 in colorectal cancer therapy.

Taken together, our results not only reveal a critical role for miR-143 and miR-145 as tumor suppressors in colorectal carcinogenesis through repression of IGF1R translation but also show that different miRNAs within a cluster can simultaneously repress a given target mRNA. This study may provide a potential novel target for future colorectal cancer therapy.

## Supporting Information

File S1 **Supporting figure and table. Tables S1.** Table S1 (in File S1) Clinical features of colorectal cancer patients. **Figure S1.** Figure S1 (in File S1) Downregulation of IGF1R by siRNA and overexpression of IGF1R by IGF1R vector. (A and B) Western blot analysis of IGF1R protein levels in Caco2 cells treated with a control siRNA and an IGF1R siRNA. A: representative image; B: quantitative analysis. (C) Quantitative RT-PCR analysis of IGF1R mRNA levels in Caco2 cells treated with a control siRNA and an IGF1R siRNA. (D and E) Western blot analysis of IGF1R protein levels in Caco2 cells treated with a control vector and an IGF1R vector. D: representative image; E: quantitative analysis. (F) Quantitative RT-PCR analysis of IGF1R mRNA levels in Caco2 cells treated with a control vector and an IGF1R vector. *P<0.05; **P<0.01.
(DOC)
