# Supplementary material for: MiR-143 and MiR-145 Regulate IGF1R to Suppress Cell Proliferation in Colorectal Cancer
Source: PLoS One. 2014 Dec 4;9(12):e114420. doi: 10.1371/journal.pone.0114420 (PMC4256231; doi:10.1371/journal.pone.0114420)

**Supporting information**

**Table S1.** Clinical features of colorectal cancer patients.

|  | Age | Gender | Pathological Stage | Tumor Histotype |
| --- | --- | --- | --- | --- |
| 1 | 62 | M | III(T3,N1,M0) | Adenocarcinoma |
| 2 | 79 | M | IV(T4,N2,M1) | Adenocarcinoma |
| 3 | 85 | F | III(T3,N1,M0) | Adenocarcinoma |
| 4 | 68 | M | II(T3,N0,M0) | Adenocarcinoma |
| 5 | 63 | M | II(T4,N0,M0) | Adenocarcinoma |
| 6 | 51 | F | III(T4,N2,M0) | Adenocarcinoma |

**Figure S1. Downregulation of IGF1R by siRNA and overexpression of IGF1R by IGF1R vector. (A and B)** Western blot analysis of IGF1R protein levels in Caco2 cells treated with a control siRNA and an IGF1R siRNA. A: representative image; B: quantitative analysis. **(C)** Quantitative RT-PCR analysis of IGF1R mRNA levels in Caco2 cells treated with a control siRNA and an IGF1R siRNA. **(D and E)** Western blot analysis of IGF1R protein levels in Caco2 cells treated with a control vector and an IGF1R vector. D: representative image; E: quantitative analysis. **(F)** Quantitative RT-PCR analysis of IGF1R mRNA levels in Caco2 cells treated with a control vector and an IGF1R vector. * P < 0.05; ** P < 0.01.


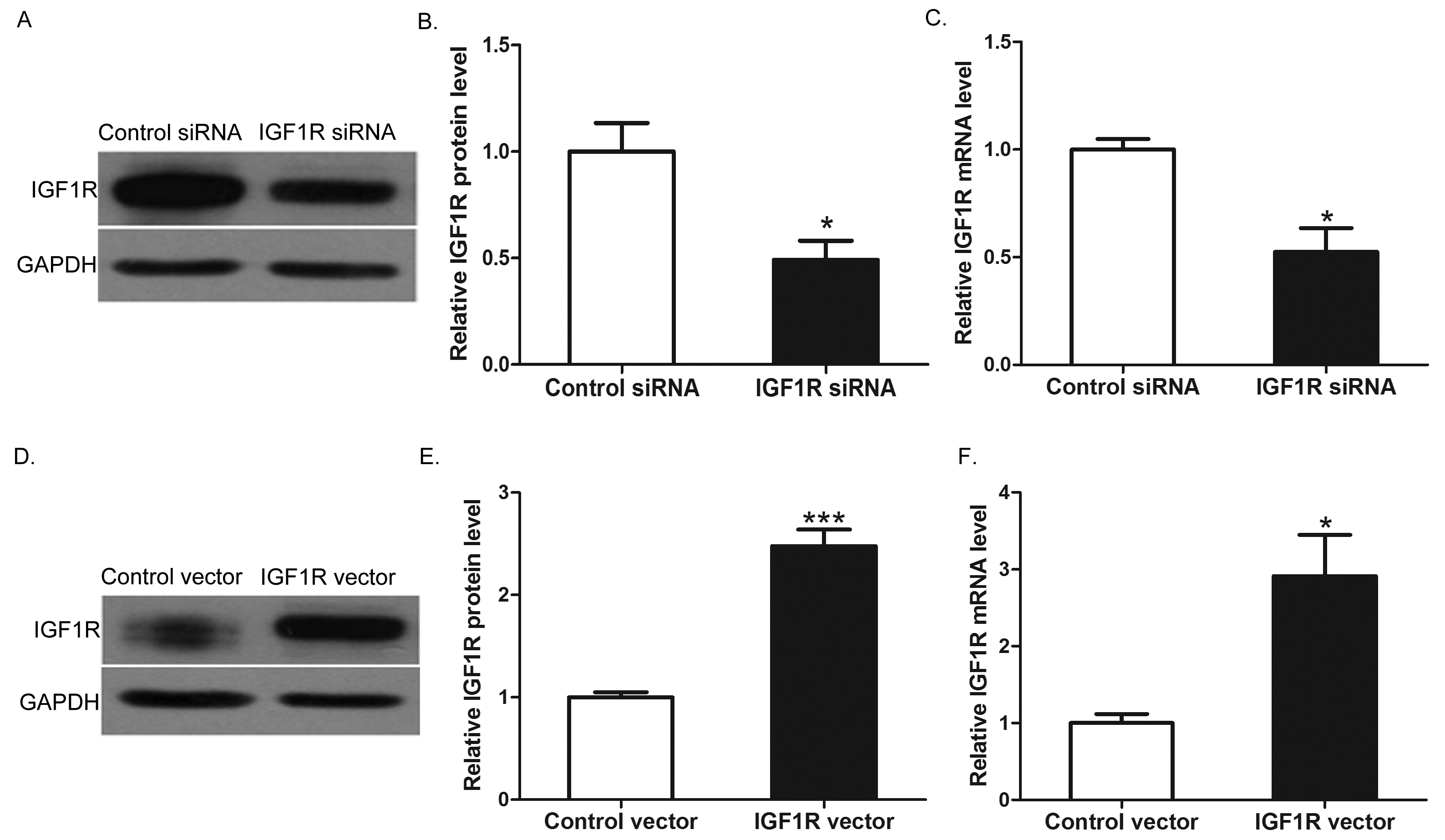

Supplement: File S1 — Supporting figure and table. Tables S1. Table S1 (in File S1) Clinical features of colorectal cancer patients. Figure S1. Figure S1 (in File S1) Downregulation of IGF1R by siRNA and overexpression of IGF1R by IGF1R vector. (A and B) Western blot analysis of IGF1R protein levels in Caco2 cells treated with a control siRNA and an IGF1R siRNA. A: representative image; B: quantitative analysis. (C) Quantitative RT-PCR analysis of IGF1R mRNA levels in Caco2 cells treated with a control siRNA and an IGF1R siRNA. (D and E) Western blot analysis of IGF1R protein levels in Caco2 cells treated with a control vector and an IGF1R vector. D: representative image; E: quantitative analysis. (F) Quantitative RT-PCR analysis of IGF1R mRNA levels in Caco2 cells treated with a control vector and an IGF1R vector. *P<0.05; **P<0.01. (DOC) [file pone.0114420.s001.doc]
